# Inspiratory High Frequency Airway Oscillation Attenuates Resistive Loaded Dyspnea and Modulates Respiratory Function in Young Healthy Individuals

**DOI:** 10.1371/journal.pone.0091291

**Published:** 2014-03-20

**Authors:** Theresa Morris, David Paul Sumners, David Andrew Green

**Affiliations:** 1 Centre of Human & Aerospace Physiological Sciences, King’s College London, London, United Kingdom; 2 Applied Science, Engineering, Science and the Built Environment, London South Bank University, London, United Kingdom; Research Center Borstel, Germany

## Abstract

Direct chest-wall percussion can reduce breathlessness in Chronic Obstructive Pulmonary Disease and respiratory function may be improved, in health and disease, by respiratory muscle training (RMT). We tested whether high-frequency airway oscillation (HFAO), a novel form of airflow oscillation generation can modulate induced dyspnoea and respiratory strength and/or patterns following 5 weeks of HFAO training (n = 20) compared to a SHAM-RMT (conventional flow-resistive RMT) device (n = 15) in healthy volunteers (13 males; aged 20–36 yrs). HFAO causes oscillations with peak-to-peak amplitude of 1 cm H_2_O, whereas the SHAM-RMT device was identical but created no pressure oscillation. Respiratory function, dyspnoea and ventilation during 3 minutes of spontaneous resting ventilation, 1 minute of maximal voluntary hyperventilation and 1 minute breathing against a moderate inspiratory resistance, were compared PRE and POST 5-weeks of training (2×30 breaths at 70% peak flow, 5 days a week). Training significantly reduced NRS dyspnoea scores during resistive loaded ventilation, both in the HFAO (p = 0.003) and SHAM-RMT (p = 0.005) groups. Maximum inspiratory static pressure (cm H_2_O) was significantly increased by HFAO training (vs. PRE; p<0.001). Maximum inspiratory dynamic pressure was increased by training in both the HFAO (vs. PRE; p<0.001) and SHAM-RMT (vs. PRE; p = 0.021) groups. Peak inspiratory flow rate (L.s^−1^) achieved during the maximum inspiratory dynamic pressure manoeuvre increased significantly POST (vs. PRE; p = 0.001) in the HFAO group only. HFAO reduced inspiratory resistive loading–induced dyspnoea and augments static and dynamic maximal respiratory manoeuvre performance in excess of flow-resistive IMT (SHAM-RMT) in healthy individuals without the respiratory discomfort associated with RMT.

## Introduction

Respiratory function is not typically seen as a limitation to activity or exercise performance in healthy individuals, although blood flow re-distribution from skeletal muscles has been suggested during near maximal workloads [1]. Activity limitation is however a feature of respiratory pathology such as Chronic Obstructive Pulmonary Disease (COPD) (e.g. see review: [2]). Compromised respiratory function (e.g. inspiratory muscle weakness; [3]) can reduce exercise tolerance, via the generation of dyspnoea [4] and the promotion of respiratory fatigue [5].

Dyspnoeic perception is a vital constituent of respiratory-induced activity limitation [6], which processes multiple dimensions: sensations of work/effort, tightness, and air hunger/unsatisfied inspiration in addition to sensory–perceptual experience, affective distress, or symptom/disease impact or burden domains that may co-exist [7]. The experience of dyspnoea can be produced when there is elevated work of breathing [8] presumably via a combination of pulmonary and extra-pulmonary afferent receptors (see [7]) and cortical motor command or corollary discharge [9] that is perceived as uncomfortable or unpleasant [10] [11] [12].

COPD is specifically associated with increased respiratory work (41) and skeletal neuromuscular weakness [13]. Such mechanisms can perpetuate a viscous cycle of functional decline (for review see [14]). Experimental elevation of the perception of breathing effort/work can be reproduced via external resistive or elastic loads [15] by volitional hyperpnoea [16], or neuromuscular blockade [17].

Respiratory muscle training (RMT) and specific inspiratory muscle training (IMT) via flow (nonlinear) resistive loading [18], or pressure-threshold loading [19] [20] has been proposed to improve respiratory function, in health and disease (e.g. [21]). IMT has been shown to increase inspiratory muscle strength, endurance, and relieve exercise-induced dyspnoea [22] [23] [24] in normal healthy individuals [19]. Various training regimes have been employed with high force-low flow IMT eliciting increased force generation (i.e. maximum inspiratory pressure) [25] [26], whereas low force-high flow training eliciting greater flow augmentation [18].

In COPD, unsupervised inspiratory resistive or threshold training at 30% of maximal static respiratory muscle strength (MIP), evoked inspiratory muscle strength, endurance and dyspnoea score improvements [27], [28]. Structural changes such as diaphragmatic thickening [22], [29], and/or increased external intercostal muscle type II fibre size [30] have been noted after only 5 weeks of IMT. However, RMT suitability as a therapy, particularly in those whom are stable remains in question [3], [31]–[33] with unpleasant exaggeration of acute dyspnoea during IMT leading to poor adherence [34] that limits clinical utility.

Short-term tendon or muscle vibration (2–20 s) has been shown to augment skeletal muscle force [35], [36], discharge rates of motor units [37] and primary afferents [38]–[40] whilst modulating spindle afferent feedback [40]. Furthermore, repeated exposure to targeted and whole body vibration (WBV) during muscle contractions, can improve strength [41] and flexibility [42], [43]. Interestingly, WBV has been shown to induce hyperventilation [44], whereas, direct chest-wall percussion can reduce breathlessness in respiratory-limited subjects [45]. However, no device for delivering inspiratory chest wall vibration is available.

High-frequency airway oscillation (HFAO), a novel form of respiratory vibratory stimulation has been shown, following a 10 breath exposure, to increase tidal and dynamic inspiratory mouth pressures (MAX_MP_) and peak inspiratory flow (PIF) [46], due to inspiratory neural drive augmentation (see review [47]). Intriguingly, neural drive changes to the respiratory accessory muscles [48] have been implicated in the development of acute dyspnoea and COPD [49], [50]. Indeed, vibration-induced augmentation of afferent feedback from the parasternal intercostal muscle spindles using vibration, reduces breathlessness in COPD [35], [45], [51]–[53], that may also, by lowering central neural drive for a given output force, represent improved respiratory mechanical efficiency [54], [55]. However, the timing and site of application of a vibratory stimulus appear important but whose optimal characteristics and form of delivery are unknown [7].

Given the augmentation of voluntary respiratory performance (reflective of increased respiratory output for maximal respiratory drive) seen in response to acute HFAO [46], it potentially offers a high flow/low force method of respiratory training without the unpleasant sensations associated with conventional forms of IMT (high force/low flow). Furthermore, given the fact HFAO appears to modulate respiratory control in a manner different to external respiratory resistance alone [56], we sought to investigate whether HFAO training has an effect upon respiratory function compared to flow-resistive RMT (SHAM-RMT device; that provides similar resistance but no oscillations) thereby addressing the common issue of uncontrolled trials [57] and possibility of placebo effects [58]. The effect of 5-weeks of training was compared during spontaneous, inspiratory resistive loaded (to model restricted respiratory flow i.e. COPD) and voluntary hyperventilatory (to reflect volitional respiratory muscle recruitment capacity) breathing, in young healthy individuals.

## Materials and Methods

### Subjects

Thirty-seven healthy volunteers (non-smokers, free from respiratory disorders; 13 males; aged 20–36 yrs.) gave written informed consent to participate in this blinded training study, approved by the King’s College London (Biomedical Sciences, Medicine, Dentistry and Natural & Mathematical Sciences) ethics committee. One subject was removed from the HFAO group as they trained with a faulty aerosure™ and another from the SHAM-RMT group due to a valve leak during the POST testing session. Hence, 20 participants (6 male, mean [±SD] age 24.2±1.8 yrs.; 170.6±8.9 cm; 68.9±12.6 kg; BMI: 23.5±2.3 kg/m^2^) were assigned to the High Frequency Oscillation (HFAO) training group and 15 participants (9 male, age 26.3±3.9 yrs.; 173.4±9.1 cm; 74.7±15.2 kg; 24.7±3.5 kg/m^2^) to the sham (SHAM-RMT) control training group. Participants were all recreationally active, were instructed to adhere to their usual activity regimen and not to engage in strenuous exercise the day before testing.

### Study Design

Respiratory function assessment was performed identically, PRE and POST a 5-week training period, with participants tested at the same time of day to minimise any diurnal variability. Participants were asked to refrain from consuming alcohol for 24 hours and caffeine for 3 hours prior to testing in addition to being at least 2 hours post-prandial. Following PRE testing, participants were blindly provided with either a fully functional HFAO (aerosure™ V1; Actegy Ltd, Ascot, UK), or SHAM-RMT device. The HFAO device incorporated a rotating (25 Hz) valve that momentarily restricts airflow causing it to oscillate with a peak-to-peak amplitude of 1 cm H_2_O, whilst providing a background resistance (during both inspiration and expiration) to flow of 21 cm H_2_O·L^−1^·s^−1^. The SHAM-RMT group received a visually and operatively identical HFAO device, except that it was fitted with a shortened rotating valve that did not occlude airflow, and thus created no pressure oscillation, whilst providing a background resistance of 20 cm H_2_O**·**L^−1^·s^−1^.

### Testing Procedures

Participants sat up straight in a comfortable but firm chair throughout testing, breathing through a mouth piece with a nose clip on. Lung volumes (Inspiratory Capacity, IC; Inspiratory Vital Capacity; IVC, Expiratory Vital Capacity; EVC, Expiratory Residual Volume; ERV) were calculated from maximal inspiratory and expiratory manoeuvres (L_STPD_; Fleisch pneumotachograph No. 4, Lausanne, Switzerland; P.K. Morgan Digital Integrator, Morgan, UK) according to the ATS/ETS guidelines [59]. In addition, maximal forced expirations: forced vital capacity (FVC) and forced expiratory volume in 1 second (FEV_1_) (L_STPD_; Vitalograph™ spirometer; Buckingham, UK) were recorded.

Participants also performed maximal dynamic inhalations from a point of maximal exhalation in which peak inspiratory flow (PIF; L·s^−1^), peak inspiratory mouth pressure (MAX_MP;_ cmH_2_O), and maximal inspiratory volume (L_STPD_) were determined. Maximal static inspiratory mouth pressure (MIP) was recorded against a bung restricting airflow (2 mm open port to prevent the participants developing elevated Buccal pressure) after a maximal exhalation. During both the MAX_MP_ and MIP manoeuvres participants were instructed to fully expire, and then take a rapid maximal inspiration and hold for approximately 4 seconds before the mouth piece was removed and they relaxed.

Breath-by-breath respiratory parameters; inspiratory flow (L·s^−1^_STPD_; Fleisch pneumotachograph No. 4, Lausanne, Switzerland), inspiratory volume (L_STPD_; Morgan digital integrator, Morgan, UK), inspiratory mouth pressure (cm H_2_O; DP45-32 pressure transducer, Validyne, California, USA) and end-tidal carbon dioxide (P_ET_CO_2_; mmHg; Servomex™ 1440 fast response Gas Analyser, Servomex™, Crowborough, UK) via polyethylene tubing from a vent located close to the mouth piece and Oxygen saturation at the ear (Ohmeda Pulse Oximeter, USA) were recorded during 3 challenge periods. The challenges were: 3 minutes of spontaneous resting ventilation (SRV), 1 minute breathing against an inspiratory resistance of 34 cm H_2_O·L^−1^·s^−1^ (Resistance loaded ventilation; RLV) provided by a narrow bore (3 mm) tubing bore (length; 100 mm), and 1 minute of maximal voluntary hyperventilation (MVH). Each challenge was followed by at least 3 minutes of recovery prior to the next phase in order to negate carry-over effects.

In the final seconds of the RLV challenge dyspnoea as indicated by ‘difficulty in breathing’ in the sensory–perceptual domain was recorded by pointing at an 11-point numeric scale (0–10) [60]. It was established that during resting spontaneous breathing was associated with a score of zero in each participant. 0 was anchored as no sensation and 10 as impossible to breathe. NRS scores were also recorded using the device allocated to that subject prior to (PRE) and following training (POST). Participant’s general wellbeing was assessed with the General Health Questionnaire, SF-12 and Modified Physical Activity Readiness Questionnaires (PAR-Q) before each testing session to ensure the absence of gross health status differences.

### Training Protocol

Participants were asked to breathe through their (HFAO or SHAM-RMT) device, with approximately 70% of their maximal inspiratory peak flow (determined and practised in the PRE testing session), 60 times per day (in 2 sets of 30 breaths), 5 days per week, for 5 consecutive weeks. All training was conducted in their own time, away from the laboratory. Each participant received a rubber mouthpiece, nose clip, user manual and were carefully instructed on how to use and care for the device they were given. In order to encourage compliance participants were provided with a daily training diary and received a weekly phone call or text message and were asked to inform the investigator of any problems encountered during the training as soon as possible.

### Data Analysis

All data apart from that from the vitalograph, questionnaire and dyspnoea ratings were sampled via an A-D converter (Powerlab, ADInstruments Ltd, Oxford, UK) at 200 Hz and stored with LabChart software (ADInstruments Ltd, Oxford, UK). Stored challenge data files were converted to Spike (v.6, Cambridge Electronic Design, Cambridge, UK) and analysed using bespoke offline scripts. Mean (±SEM) values were calculated for the respiratory parameters recorded during the final 2 minutes of SRV and the entire 1 minute of RES and MVH. The greatest achieved values were used for all lung function measures having performed each manoeuvre on at least 3 occasions. All physiological parameters were normally distributed (Kolmogorov-Smirnov 1-sample *t*-test; SPSS v16, SPSS Inc., Chicago, IL), hence 2-way ANOVAs (time-by-group) were performed to investigate the effect of 5-weeks of training (time effect) with the interaction (time*group) effect indicative of differential responses (PRE vs. POST) in the HFAO and SHAM-RMT groups. Post-hoc *t*-tests were performed for each group where time or interaction effects were noted to determine the location of effect (PRE vs. POST). If group effects were noted, post-hoc independent samples t-tests were performed on PRE or POST intervention (SHAM-RMT vs. HFAO) data. Dyspnoea scores in response to RVH were compared PRE vs. POST 5-weeks training for each group with a Mann-Whitney U test. To investigate if the increase in maximal respiratory muscle performance was responsible for the reductions in dyspnoea, linear regression analysis was conducted between maximal static (MIP) and dynamic (MAX_MP_) respiratory performance, peak inspiratory flow (PIF) and HFAO-induced reduction in breathlessness. In all cases statistical significance was defined as p<0.05.

## Results

The only significant demographic difference between groups was the greater proportion of males allocated to the HFAO group (χ^2^ = 4.263, p = 0.039). Each participant reported compliance with the training protocol whilst stating overall physical activity was unchanged for the duration of the study. None of the participants were aware there was a control group and all returned for their allotted post-testing session without comment.

### Lung Volumes

There were no significant changes in IC, IVC, EVC or ERV following 5-weeks training in either group (Table 1). Training produced a small, albeit statistically significant reduction in FVC (L_STPD_) [F(1,31) = 7.266; p = 0.011] across the groups (time effect). However, an interaction effect [F(1,31) = 4.014, p = 0.054] trend highlighted a significant reduction in the SHAM-RMT (p<0.001), but not the HFAO group. Similarly, a significant reduction in FEV_1_ (L_STPD_) was noted across groups following training [F(1,31) = 13.946, p = 0.001]. A significant interaction effect: [F(1,31) = 7.737; p = 0.009] was located by post-hoc testing to a reduction in the SHAM-RMT (p = 0.004) group. SHAM-RMT FVC and FEV_1_ reductions combined to render FEV_1_/FVC (%) unchanged, as was expiratory peak flow (L.s^−1^) in either group (Table 1).

**Table 1:** Mean (±SEM) lung function PRE and POST 5 weeks of training in the SHAM-RMT and HFAO groups.

	SHAM-RMT		HFAO	
	PRE	POST	PRE	POST
**IC (L)**	2.77±0.17	2.73±0.15	2.52±0.16	2.52±0.16
**IVC (L)**	4.09±0.25	4.03±0.25	3.69±0.22	3.65±0.23
**EVC (L)**	4.13±0.26	3.84±0.32	3.30±0.28	3.72±0.23
**ERV (L)**	1.40±0.13	1.33±0.13	1.21±0.09	1.16±0.10
**FVC (L)**	4.40±0.26	4.26±0.26*	3.9±0.26	3.88±0.25
**FEV_1_ (L.s^−1^)**	3.66±0.21	3.49±0.22*	3.2±0.18	3.17±0.18
**FEV_1_/FVC (%)**	83.37±1.49	82.02±1.45	82.90±1.48	82.39±1.25
**Insp Flow (L.s^−1^)**	6.08±2.66	6.03±2.93	5.33±2.34	5.31±2.29

### Respiratory Muscle Strength

**Figure 1:**
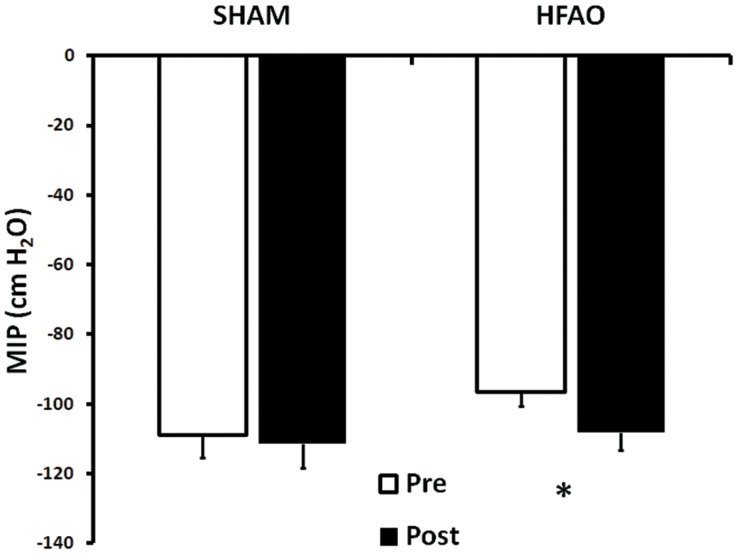
Mean (±SEM) Maximal Static Inspiratory Pressure (MIP; cm H_2_O), PRE and POST 5 weeks of training in the SHAM-RMT and HFAO groups. * Indicates significant difference PRE vs. POST for that group (p<0.05).

**Figure 2:**
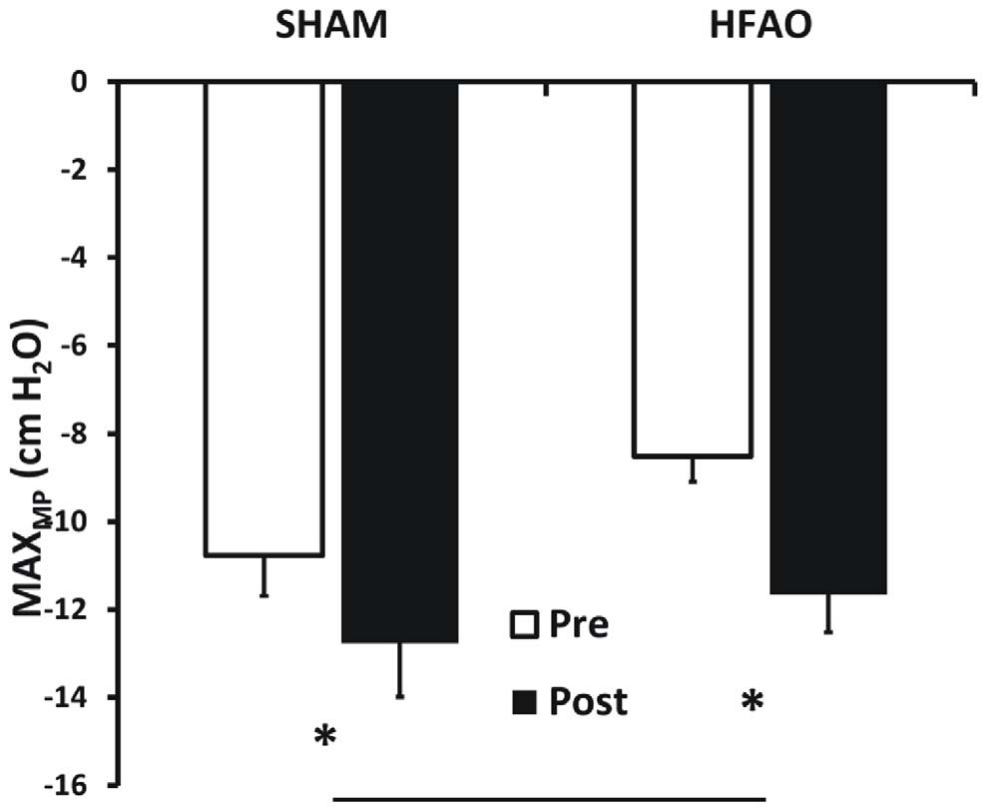
Mean (±SEM) Maximal Dynamic Inspiratory Pressure (MAX_MP_; cm H_2_O), PRE and POST 5 weeks of training in the SHAM-RMT and HFAO groups. * Indicates significant difference PRE vs. POST for that group whilst the solid bar indicates significant difference at PRE (SHAM-RMT vs. HFAO) (p<0.05).

MIP values were significantly lower in the HFAO group PRE (vs. SHAM-RMT; p = 0.037) but not different POST training. Hence, 5-weeks of training significantly increased MIP (cm H_2_O)[F(1,31) = 12.564; p = 0.001], in the HFAO group only (Figure 1; −108.96 v −111.41, Pre v Post, SHAM; −96.56 v −108.04, PRE v POST, HFAO; interaction effect [F(1,31) = 5.277; p = 0.029]). MAX_MP_ (cm H_2_O) was increased by training [F(1,31) = 21.272; p = 0.001] in both the HFAO (vs. PRE; p<0.001) and SHAM-RMT (vs. PRE; p = 0.021) (Figure 2) groups.

**Figure 3:**
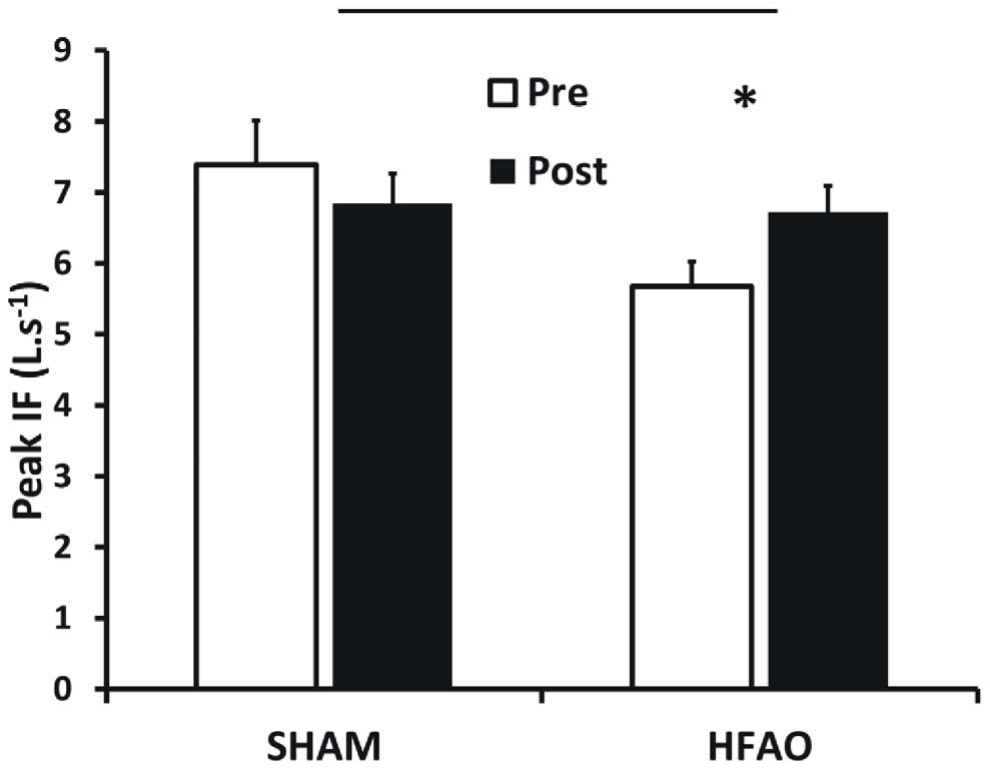
Mean (±SEM) Peak Inspiratory Flow (PIF; L.s^−1^), PRE and POST 5 weeks of training in the SHAM-RMT and HFAO groups. * Indicates significant difference PRE vs. POST for that group (p<0.05).

Peak inspiratory flow rate (PIF; L.s^−1^) was significantly lower in the HFAO group PRE (vs. SHAM-RMT; p = 0.016) but there was no difference POST training. PIF achieved during the MAX_MP_ manoeuvre did not increase significantly across groups. However, an interaction effect [F(1,31) = 11.625; p = 0.002] highlighted an increase POST (vs. PRE; p = 0.001) in the HFAO group, contrasting with a non-significant decrease in the SHAM-RMT group (Figure 3).

### Spontaneous Resting Ventilation (SRV)

Spontaneous resting expiratory time (T_E_; s) and respiratory frequency (f_R_: min^−1^), were unchanged. In contrast, inspiratory time (T_I_; s) had a tendency to shorten following training (PRE vs. POST) [F(1,31) = 4.008; p = 0.054](Table 2). T_I_ shortening was limited to the HFAO group (p = 0.017), rendering SHAM-RMT significantly longer POST (p = 0.044). Mean peak inspiratory mouth pressure (Peak IMP; mmHg) was reduced following training [F(1,31) = 10.133; p = 0.003] albeit significant only in the SHAM-RMT group (vs. PRE; p = 0.005) (interaction effect; [F(1,31) = 3.816; p = 0.006]). In fact, peak IMP both PRE (vs. SHAM-RMT; p = 0.004) and POST (p<0.001) were significantly greater in the HFAO group.

**Table 2:** Mean (±SEM) breath-by-breath parameters during spontaneous resting ventilation (SRV), PRE and POST 5 weeks of training in the SHAM-RMT and HFAO groups.

Spontaneous Resting Ventilation (SRV)	SHAM-RMT		HFAO	
	PRE	POST	PRE	POST
**T_I_ (s)**	2.1±0.2	2.1±0.2	1.9±0.1	1.6±0.1*#
**T_E_ (s)**	3.7±0.4	3.6±0.4	3.2±0.3	3.1±0.3
**fR (min^−1^)**	10.4±1.1	10.5±1.0	11.9±1.2	12.9±1.2
**Peak IMP (cm H_2_O)**	−1.6±0.1	−1.2±0.1*	−1.8±0.1#	−1.7±0.1#
**Peak Insp Flow (L.s^−1^)**	0.52±0.02	0.61±0.03*	0.60±0.04	0.64±0.04
**V_T_ (mL_STPD_)**	0.72±0.05	0.84±0.05	0.74±0.07	0.61±0.07
**V_I_ (L_STPD_.min^−1^)**	6.70±0.57	7.8±0.44 *	7.8±0.63	8.07±0.61

* Indicates significant difference PRE vs. POST for that group (p<0.05).

# Denotes a significant difference PRE or POST (SHAM-RMT vs. HFAO) (p<0.05).

Significant increments of resting peak inspiratory flow (PIF; L_STPD_.s^−1^) were observed, across both groups [F(1,31) = 6.602; p = 0.015], but post-hoc tests revealed that these achieved statistical significance in the SHAM-RMT group only (vs. PRE; p = 0.018). Spontaneous tidal volume (V_T_; mL_STPD_) was significantly increased in the SHAM-RMT group (p = 0.003), whilst remaining unchanged in the HFAO group (interaction effect; [F(1,31) = 4.051; p = 0.053]). In contrast, minute ventilation (V_I_; L_STPD_.min^−1^) significantly increased over time [F(1,31) = 4.431, p = 0.044], although only significant in the SHAM-RMT group (p = 0.015).

### Voluntary Hyperventilation (VHV)

T_I_, T_E_ and f_R_ were unchanged during VHV in either group following training. Peak IMP [F(1,31) = 11.335; p = 0.002] was augmented across groups, being significant POST (vs. PRE) for both HFAO (p = 0.019) and SHAM-RMT (p = 0.048) (Table 3). The SHAM-RMT group had significantly greater Peak IMP, both PRE (p = 0.050) and POST (p = 0.002). PIF was significantly augmented (interaction: [F(1,31) = 5.180; p = 0.030] in the HFAO group only (p = 0.032), with HFAO PIF significantly lower PRE (p = 0.003) but not POST. 5-weeks of training induced increments in V_T_ [F(1,31) = 5.053; p = 0.032] and V_I_ [F(1,31) = 4.511; p = 0.042] rendering HFAO group values that were lower PRE (p = 0.009 and p = 0.014 respectively) but not different POST.

**Table 3:** Mean (±SEM) breath-by-breath respiratory parameters during voluntary hyperventilation (VHV), PRE and POST 5 weeks of training in the SHAM-RMT and HFAO groups.

Voluntary Hyperventilation (VHV)	SHAM-RMT		HFAO	
	PRE	POST	PRE	POST
**T_I_ (s)**	0.6±0.1	0.6±0.1	0.5±0.0	0.5±0.0
**T_E_ (s)**	0.6±0.1	0.6±0.1	0.5±0.0	0.6±0.0
**fR(min^−1^)**	52.2±5.3	49.2±6.5	57.7±4.4	55.0±6.1
**Peak IMP (cm H_2_O)**	−7.2±0.4	−8.2±0.8*	−5.7±0.4#	−6.5±0.5*#
**Peak Insp Flow (L.s^−1^)**	5.07±0.38	4.83±0.40	3.52±0.30#	3.95±0.34*
**V_T_ (mL_STPD_)**	1.69±0.16	1.79±0.16*	1.32±0.14	1.40±0.18*
**V_I_ (L_STPD_.min^−1^)**	9.03±0.86	8.84±0.93	7.27±0.62#	7.82±0.69#*

* Indicates significant difference PRE vs. POST for that group (p<0.05).

# Denotes a significant difference PRE or POST (SHAM-RMT vs. HFAO) (p<0.05).

### Resistive Loaded Ventilation (RLV)

Inspiratory resistive loaded T_I_, T_E_, f_R_, were unchanged in either group following 5–weeks training (Table 4). Peak IMP was significantly augmented across both groups [F(1,31) = 5.725; p = 0.023] following training, but post-hoc individual group testing was non-significant. Significant increments in PIF [F(1,31) = 11.796, p = 0.002] were observed in the SHAM-RMT (p = 0.002) group only (interaction effect; [F(1,31) = 11.146; p = 0.002]).

**Table 4:** Mean (±SEM) breath-by-breath respiratory parameters during resistive loaded ventilation (RLV), PRE and POST 5 weeks of training in the SHAM-RMT and HFAO groups.

Resistive Loaded Ventilation (RLV)	SHAM-RMT		HFAO	
	PRE	POST	PRE	POST
**T_I_ (s)**	4.4±0.6	4.2±0.5	3.2±0.3	3.0±0.3
**T_E_ (s)**	2.7±0.3	2.4±0.3	2.1±0.2	2.1±0.3
**fR (min^−1^)**	8.4±1.6	9.1±1.5	11.4±1.2	11.7±1.3
**Peak IMP (cm H_2_O)**	−16.4±1.2	−21.9±3.4	−16.5±1.4	−17.3±1.9
**Peak Insp Flow (L.s^−1^)**	0.24±0.01	0.31±0.02*	0.27±0.02	0.27±0.01
**V_T_ (mL_STPD_)**	0.75±0.08	0.96±0.11*	0.61±0.07	0.52±0.06#
**V_I_ (L_STPD_.min^−1^)**	5.70±0.44	7.82±0.78*	6.29±0.27	6.06±0.28#

* Indicates significant difference PRE vs. POST for that group (p<0.05).

# Denotes a significant difference PRE or POST (SHAM-RMT vs. HFAO) (p<0.05).

V_T_ increased only in the SHAM-RMT group (interaction effect; [F(1,31) = 6.840; p = 0.014]), whilst remaining unchanged in the HFAO group. This contributed to (group effect; [F(1,31) = 7.094; p = 0.012]) SHAM-RMT being significantly greater than HFAO POST training (p = 0.002). V_I_ increased across the groups [F(1,31) = 11.759; p = 0.002], however an interaction effect [F(1,31) = 11.146; p = 0.002] and post-hoc testing indicated increments were only in the SHAM-RMT group (p = 0.002). Oxygen saturation was unchanged (97% ±2) with respect to SRV (98% ±2).

### Subjective Respiratory Function - Dyspnoea

**Figure 4:**
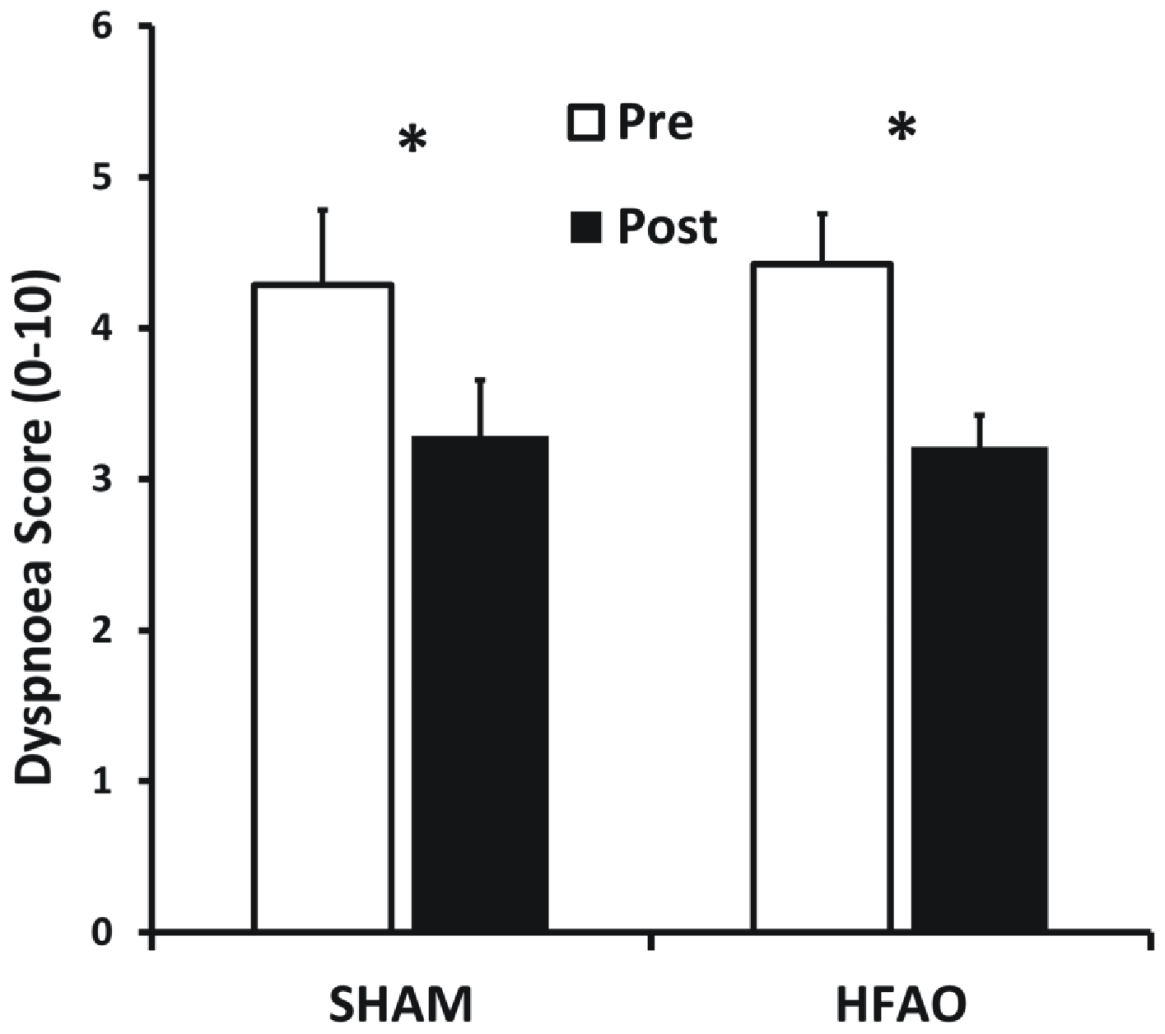
Mean (±SEM) Dyspnoea during Resistive Loaded Ventilation (RLV; NRS Score; 0–10), PRE and POST 5 weeks of training in the SHAM-RMT and HFAO groups. * Indicates significant difference PRE vs. POST for that group (p<0.05).

NRS dyspnoea scores during inspiratory resistance loading were significantly reduced, both in the HFAO (p = 0.003) and SHAM-RMT (p = 0.005) groups following training (Figure 4). NRS scores of 0 were declared during HFAO and SHAM-RMT use PRE, during and POST training. There was no correlation between improvements in MIP, MAX_MP_ or PIF and reduced dyspnoea.

## Discussion

The main findings of this study were that 5-weeks high-frequency airway oscillation (HFAO) training caused a significant reduction in dyspnoea during an inspiratory resistive loaded challenge and significant increase in peak inspiratory airflow (PIF) during a maximal inhalation. This was accompanied by an augmentation in both maximal dynamic (MAX_MP_) and static inspiratory pressure development (MIP) but only a strong tendency towards increased peak mouth pressure development during maximal voluntary hyperventilation. HFAO training had no effect upon ventilation during spontaneous nor during an inspiratory resistive loading challenge. Some of the effects seen following HFAO training were also observed after SHAM-RMT training highlighting the need for appropriate placebo control groups.

Both HFAO and SHAM-RMT training relieved dyspnoea during an inspiratory resistive-loaded challenge, albeit the former was slightly greater. A reduction of 1 on a 0–10 NRS (or its equivalent on other ratings scales e.g. visual analogue [61], BORG [62], Likert-like [63] scales is functionally significant [64] in excess of the minimally clinically important difference (MCID) irrespective of the cause of dyspnoea [65]–[67].

IMT has been shown to reduce dyspnoea, although the mechanism(s) by which this is achieved is unclear [28]. Reduction may reflect improved respiratory muscle efficiency [68] and/or augmented inspiratory muscle force generation capacity that would lower efferent drive required for a given V_I_ [54], [69]. Presumably this would attenuate corollary discharge to the respiratory sensory areas [70], thereby limiting dyspnoea [71], [72]. IMT may also reduce accessory respiratory muscle recruitment, which appears to enhance dyspnoeic sensations more strongly than elevated diaphragmatic activation [68].

Unfortunately, this study did not measure respiratory EMG [48] and thus cannot assess changes in activity nor estimate diaphragmatic/accessory respiratory muscle balance [73]. In addition, measurement of lung volume and thoracic-abdominal motions with magnetometry might inform whether length-tension diaphragmatic advantages are elicited [74] which may reduce the work of breathing, dyspnoea [75], inependent of vagal-induced modulation [76] and changes in alveolar/arterial blood gas levels [77]. However, regression analysis revealed that the relief of dyspnoea during RLV was unrelated to increase in performance during maximal respiratory maneuvers, suggesting that the mechansisms behind reduced dyspnoea and improved respiratory strength differ. This counters the hypothesis that increased force generating capacity lowers corollary discharge [78] for a given ventilatory load, but mirrors the variable relationship between FEV_1_ and dyspnoea in provocated asthma [79].

Respiratory vibration appears to have phase-specific effects as in-phase chest wall vibration during inspiration relieved breathlessness [45], whereas out of phase vibration increased breathlessness [51] in COPD. Thus, muscle spindle reflex activation modifies the cortico-spinal respiratory controller input-output relationship – reducing central respiratory drive [80]–[82] and may underlie HFAO–induced dyspnoea relief and acute maximal respiratory augmentation [46]. Such an effect would be particularly advantageous in COPD patients as neural respiratory drive is elevated and appears related to disease severity [48] and has been suggested as a potential approach for intractable dyspnoea [83]. However, this would contradict data showing no supraspinal effect of canine rib vibration [84]. In fact the role of chest wall mechanoreceptor feedback in providing relief is equivocal [85]. Further, because respiratory muscle EMG was not recorded in this study, it is difficult to speculate on whether the pressure oscillations in the air column are large enough to be detected by respiratory muscle spindles or other chest wall receptors. Unpublished pilot respiratory EMG data from our lab shows harmonics around the primary oscillatory frequency suggestive of the possibility of a role for chest wall afferents.

Whether vibration-induced reductions in breathlessness are due to attenuated central respiratory drive remains to be determined, however changes in neural respiratory drive might explain the increase in ventilation (flow and pressures) observed post training. However, such effects were seen in both groups during the voluntary hyperventilation challenge. Unfortunately, dyspnoea was not recorded and isocapnia not maintained (although subjects oxygen saturation did not deviate from normal levels [∼98%]). Thus inhibitory drive [86] may have masked differential respiratory muscle performance augmentation and/or changes in feedback produced by both training stimuli such as lower lactate production seen following IMT [87], [88], attributed to increased inspiratory muscle oxidative [89] and lactate transport capacity.

HFAO-induced increments in PIF during maximal inspiratory manoeuvres suggests that performance improvements were not due to large airway bronchoconstriction, but rather that there could be airway stretch receptor mediated bronchodilation [90]–[92] that may be beneficial for airway restrictive conditions such as asthma and COPD [93]. In contrast, SHAM-RMT training saw a non-significant reduction in PIF despite an increase in MAX_mp_ which could indicate airway narrowing and increased resistance to flow [94] which is supported by the reduction in FEV_1_ and FVC in the SHAM-RMT group.

MAX_mp_ was significantly increased following HFAO, consistent with it being a high flow/low force training stimulus. SHAM-RMT also significantly increased MAX_mp_ but to a lesser extent, which may relate to the fact that the resistance provided is a flow-resistive form of RMT shown to increase respiratory muscle strength [58], [95]. The augmentation of MIP following HFAO training is comparable to other forms of IMT such as pressure-threshold training [58] but was not anticipated as HFAO is a high flow/low force mode of respiratory training. Such findings suggest that HFAO offers a broad stimulus sufficient to activate adaptive pathways that could involve improved neuromuscular recruitment, despite IMT demonstrating specificity analogous to skeletal muscle training [96]. The preservation of such effects post training was not tested but presumably is similar to the reversibility of IMT effects seen in healthy [96] and COPD patients [97], [98].

Whilst as expected HFAO had no effect on lung function in young healthy individuals, the SHAM-RMT group demonstrated a reduction in FVC and FEV_1_ that could relate to increased airway resistance [99], [100] as suggested by the increase in MAX_mp_ and reduction in PIF. However, other forms of IMT have either demonstrated no change [101] or an increase in lung function both in healthy participants and cystic fibrosis patients [102]. Whereas, the flutter expiratory mucus clearance device augmented forced vital capacity in healthy older individuals [103] suggestive that high frequency expiratory oscillations may augment respiratory performance. Increasing expiratory muscle strength could assist in reducing COPD hyperinflation [104] and strengthen cough, which may facilitate sputum expectoration [105]. Thus, HFAO might be beneficial without the promotion of dynamic hyperinflation.

In conclusion, high-frequency airway oscillation augments static and dynamic maximal respiratory manoeuvre performance in a manner comparable with conventional pressure-threshold or flow-resistive inspiratory muscle training and reduces inspiratory resistive loading–induced dyspnoea in healthy individuals without respiratory discomfort associated with training exposure. HFAO may assist elucidation of the mechanisms underlying dyspnoea and assist definition of optimal vibration characteristics for its relief.
